# CML20, an *Arabidopsis* Calmodulin-like Protein, Negatively Regulates Guard Cell ABA Signaling and Drought Stress Tolerance

**DOI:** 10.3389/fpls.2017.00824

**Published:** 2017-05-23

**Authors:** Xiaomeng Wu, Zhu Qiao, Huiping Liu, Biswa R. Acharya, Chunlong Li, Wei Zhang

**Affiliations:** ^1^Key Laboratory of Plant Cell Engineering and Germplasm Innovation, Ministry of Education, School of Life Science, Shandong UniversityJinan, China; ^2^Donald Danforth Plant Science Center, St. LouisMO, United States; ^3^College of Life Science, Jiangsu Normal UniversityXuzhou, China

**Keywords:** CML20, drought stress, guard cell, stomatal movement, abscisic acid

## Abstract

Guard cells shrink in response to drought and abscisic acid (ABA), which is caused by efflux of ions that in turn reduces stomatal aperture and improves the plant’s ability to retain moisture. Cytosolic free calcium is an essential secondary messenger in guard cell ABA signaling, but the details of this regulatory pathway remain sketchy. Here, the calmodulin-like protein CML20, which has four EF-hand domains and calcium-binding activity *in vitro*, was found to be a negative regulator of ABA-induced stomatal movement in *Arabidopsis*. The guard cells of *cml20* loss-of-function mutant plants were hypersensitive to both ABA-activated S-type anion currents, and ABA inhibited inward K^+^ currents than those of wild type. Additional, due to smaller stomatal aperture, *cml20* showed less water loss from the leaves than wild type. These phenotypes of *CML20* overexpressing plants contrasted with wild type in the opposite direction. In the *cml20* mutant, the transcripts of stress responsive genes, such as *MYB2, RAB18, ERD10, COR47*, and *RD29A* were up-regulated in response to drought and ABA, while down-regulated of *APX2* transcription and higher reactive oxygen species (ROS) accumulation. These observations support the CML20, a functional Ca^2+^ sensor, is a negative regulator in guard cell ABA signaling.

## Introduction

Drought stress represents one of the most important constraints on crop productivity. Stomatal closure is a key early plant response to drought stress (Verslues et al., 2006), a process which is controlled by the turgor of the pair of guard cells surrounding each stoma (Kwak et al., 2008). Osmotically driven influx of water is required to expand the guard cell. Drought stress promotes the tissue content of the phytohormone abscisic acid (ABA), which acts as a prominent regulator of stomatal movement through its effect on ion channel activity (Schroeder et al., 2001). ABA inhibits the inward K^+^ channel and activates the anion channel (Schroeder et al., 1987; Pandey et al., 2007; Vahisalu et al., 2008; Li et al., 2014). The major transducer of ABA signaling is free calcium ion (Ca^2+^) concentration alterations in the cytoplasm (McAinsh et al., 1990, 1997). The supply of exogenous Ca^2+^ not only can induce stomatal closure but also can oscillate the concentration of cytosolic Ca^2+^ (McAinsh et al., 1995; Pei et al., 2000; Siegel et al., 2009). Certain stimuli that induce stomatal opening may also act to enhance the concentration of cytosolic Ca^2+^ (Kenichiro et al., 1992; Cousson and Vavasseur, 1998).

Drought stress (along with other abiotic stresses) alters the plant’s cellular redox balance, often promotes the accumulation of reactive oxygen species (ROS) (Rentel and Knight, 2004). The most prominent ROS is H_2_O_2_; its prolonged half-life and high permeability allow it to activate Ca^2+^ permeable channels (Rentel and Knight, 2004). As mentioned above, stomatal movements are also associated with guard cell calcium concentration changes (Pei et al., 2000; Thor and Peiter, 2014). Ca^2+^ is required for oxidative burst by activating NADPH oxidase (Xing et al., 1997) and for the activity of NAD kinase, which generates the NADP that subsequently used as a cofactor for NADPH oxidase (Karita et al., 2004; Turner et al., 2004). Many proteins involved in ROS signaling was regulated by Ca^2+^ signaling (Gong et al., 1997; Yang and Poovaiah, 2002; Takahashi et al., 2011; Ma et al., 2012; Wang et al., 2015). Both, Ca^2+^ and ROS serve as important signaling molecules in plants, are inter-regulated by feedback loops to keep their homeostasis in plant cells (Sierla et al., 2016).

Ca^2+^ plays important signaling roles in response to abiotic stress and many Ca^2+^ sensors are targets of Ca^2+^ in the signaling pathways, and kinds of Ca^2+^ sensors have been classified into four distinct groups, namely the calmodulins (CaMs), the CaM-like proteins (CMLs), the Ca^2+^ dependent protein kinase (CDPKs), and the calcineurin B-like proteins (CBLs). All of these proteins contain EF-hand motifs, which allow the binding of Ca^2+^ (David and Anthony, 2000; Oliver and Jorg, 2012). Transcriptomic analyses have shown that some CMLs members are induced by stress (McCormack et al., 2005). Previously reports have shown that CML9 (Tzahi et al., 1999; Helen and Marc, 2002; Hongtao et al., 2003; Fabienne et al., 2008), CML18, CaM15, and CML24 (Delk et al., 2005; Yamaguchi et al., 2005) are involved in abiotic stress signaling. The function of other CMLs, which represent the largest of these four protein families, remains unclear. To understand more details about the roles of CMLs in abiotic stress signaling, we have screened and found that the mutant *cml20* is more tolerant to drought stress. As CML20 an expected Ca^2+^ sensor, we also analyzed its molecular roles in drought stress and ABA signaling pathway, especially in stomatal movement. CML20 like-proteins have been (a.k.a. CEN1) found to be present in the animal centrosome, where its role implicated in microtubule organization (Juliette et al., 2008). Here, we study the molecular role of CML20 in *Arabidopsis* in response to drought stress, and its contribution to stomatal movement, ROS production, and ion channel activity in response to ABA.

## Materials and Methods

### Plant Materials and Growth Conditions

*Arabidopsis thaliana* ecotype Columbia-0 (Col-0) and the T-DNA insertion mutant *cml20* (SALK_079974c) generated in a Col-0 background were obtained from the ABRC^1^. The zygosity of the mutant individuals was validated by PCR using the *cml20*-specific primer pair cml20-LP/-RP and the T-DNA left border primer LBb1.3^2^. The relevant primer sequences are given in **Table 1**. To grow plants, seeds were surface-sterilized by immersing in 75% v/v ethanol for 3 min, followed by 95% v/v ethanol for 1 min, then the ethanol was allowed to evaporate. The seeds were then plated on solidified (0.7% w/v agar) half strength Murashige and Skoog (1962) medium (1/2 MS) and held first for 2 days at 4°C and then kept in a growth chamber under long-day conditions (16 h light/8 h darkness, 22 ± 1/16 ± 4°C) with illumination by 100 μmol m^-2^s^-1^ light); the relative humidity was maintained at ∼70%.

**Table 1:** Primer sequences employed.

Name	Forward (5′–3′)
cml20-LP	TAGATGATGATGTGCGCAGAG
cml20-RP	AGGGTTCCATGATTGAAGAAG
LBb1.3	ATTTTGCCGATTTCGGAAC
CML20-HisF	GCCATATGATGTCGAGTATATACAGAAC
CML20-HisR	CCGCTCGAGTCACTTTGCCATCATGACTTTGAC
CaM7-HisF	GGAATTCCATATGATGGCGGATCAGCTAACCGAT
CaM7-HisR	CCGCTCGAGTCACTTTGCCATCATGACTTTGAC
CML20-OEF	GCTCTAGAATGTCGAGTATATACAGAAC
CML20-OER	GTGAGCTCCTAGTTACCACCATAAGC
CML20-PF	GTAAGCTTTCCGGAACAGGGTATGTA
CML20-PR	GAGGATCCATCTTCTACGAGTCCTCC
CML20-GFPF	GCGGATCCATGTCGAGTATATACAGAAC
CML20-GFPR	GAGTCGACGTTACCACCATAAGCAG
CML20-RTF	ATGTCGAGTATATACAGAA
CML20-RTR	CGTTACCACCATAAGCAG
ACTIN2-RTF	TCTTCTTCCGCTCTTTCTTTCC
ACTIN2-RTR	TCTTACAATTTCCCGCTCTGC
ACTIN2-QF	GGTAACATTGTGCTCAGTGGTGG
ACTIN2-QR	AACGACCTTAATCTTCATGCTGC
CML20-QF	CGCATGGCAAAGGACTTGGGT
CML20-QR	CCATCACGGTCTCGGTCTGCT
RAB18F	CGAATGGCATCCTTTCTCAATC
RAB18R	GTCACCGAGAGTGCGGATATG
COR47F	CTTGGCATTGGTGCAACTCC
COR47R	TCTTTCGTCTTGGCGTGTCA
MYB2F	TGCTCGTTGGAACCACATCG
MYB2R	ACCACCTATTGCCCCAAAGAGA
ERD10F	TCTCTGAACCAGAGTCGTTT
ERD10R	CTTCTTCTCACCGTCTTCAC
KIN1F	ACCAACAAGAATGCCTTCCA
KIN1R	CCGCATCCGATACACTCTTT
RD29F	TAATCGGAAGACACGACAGG
RD29R	GATGTTTAGGAAAGTAAAGGCTAG
KIN2F	ACCAACAAGAATGCCTTCCA
KIN2R	ACTGCCGCATCCGATATACT
APX2F	TGATGTGAAGACGAAGACAGGAGGAC
APX2R	CCCATCCGACCAAACACATCTCTTA
CML20-CF	GGGGACAAGTTTGTACAAAAAAGCAGGCTTCATGTCGAGTATATACAG
CML20-CR	GGGGACCACTTTGTACAAGAAAGCTGGGTCCTAGTTACCACCATAAGC

### Heterologous Expression of CML20 and Ca^2+^-Binding Assay

The coding region of *CML20* (*At3g50360*) was amplified from Col-0 cDNA by using the primer pair CML20-HisF/-HisR (**Table 1**), while *CaM7* (*At3g43810*) was amplified by using CaM7-HisF/-HisR (**Table 1**) as positive control. The amplicons and the prokaryotic expression vector pET30a (+) were both digested with *NdeI* and *XhoI*, and then ligated using T4 DNA ligase (TransGen Biotech, China). For heterologous expression of CML20, *E. coli* BL21 (DE3) transformants harboring recombinant plasmid *CML20* were induced with 0.4 mM isopropyl β-D-1-thiogalactopyranoside (IPTG); the recombinant protein was thereafter purified using the Ni-NTA Purification System (Invitrogen, Uinted States). Sodium dodecyl sulfate polyacrylamide gel electrophoresis (SDS-PAGE) mobility shift assay for Ca^2+^ binding was performed by exposing denatured CML20 to either 15 mM CaCl_2_ or 15 mM ethylene glycol tetraacetic acid (EGTA), as described by Garrigos et al. (1991). The structure of CML20 was predicted using InterPro software^3^.

### Construction of Plant Expression Vector and Generation of Transgenic *Arabidopsis thaliana*

To engineer plant expression vector for *CML20* over-expression, the gene’s open reading frame was amplified using the primer pair CML20-OEF/-OER (**Table 1**), and the resulting amplicon was digested with *XbaI* and *SacI*, then ligated into the *Xba*I and *Sac*I cloning sites of pSTART (+) which generated the *p35S::CML20* construct. The *CML20* open reading frame was amplified using the primer pair CML20-CF/-CR (**Table 1**) cloned into pDonor 221 vector and then introduced into pB2GW7.0 vector through gateway reaction (Invitrogen, United States) to generate pB2GW7.0-CML20. *cml20* mutant plants were transformed with pB2GW7.0-CML20 to generate complemented lines, C-1 and C-2. The *CML20* promoter sequence was amplified using the primer pair CML20-PF/-PR (**Table 1**), then ligated into the *Hin*dIII/*Bam*HI cloning site of *pCambia::UBI-GUS* to generate the construct *pCML20::GUS.* All the constructs were transferred into *Agrobacterium tumefaciens* strain GV3101 and then introduced into *Arabidopsis* using the floral dip method (Clough and Bent, 1998). T_1_ transgenic seedlings were selected by growing on 1/2 MS containing 30 mg/L kanamycin (*p35S::CML20* lines), or hygromycin (*pCML20::GUS* lines). The zygosity of T_3_ lines was deduced from the transgene’s segregation behavior, and the abundance of the transgene transcript was assessed by using PCR or quantitative real time PCR (qRT-PCR), and the selected seeds from T_3_ homozygous lines were used for further analysis.

### Subcellular Localization of CML20

A *p35S::CML20-GFP* construct was generated by inserting the *CML20* open reading frame (amplified using CML20-GFPF/-GFPR, see **Table 1**) into the *Bam*HI/*Sal*I cloning sites of a modified GFP expression vector (Lin et al., 2009). Both the *p35S::CML20*-*GFP* and the empty *p35S::GFP* plasmids were purified using a NucleoBond^®^ Xtra Midi Kit^4^ (Macherey–Nagel) before introducing into *Arabidopsis* mesophyll protoplasts following Sheen (2001). After 16 h incubation at 23°C in the dark, protoplasts were examined for GFP signal using confocal laser scanning microscopy (performed at The Microscopy Characterization Facility, Shandong University).

### GUS Staining

T_3_ homozygous transgenic plants harboring *pCML20::GUS* were assayed for GUS activity as described by Jefferson et al. (1987) with slight modification. Briefly, transgenic plant tissues were soaked in a GUS staining solution (2 mM X-Gluc, 2 mM K_3_Fe(CN)_6_, 2 mM K_4_Fe(CN)_6_, 0.1% Triton X-100, 10 mM EDTA in 50 mM sodium phosphate buffer, pH7.2) and incubated overnight at 37°C. After staining, the samples were washed in 50, 70, and 100% ethanol for 5 min consecutively, and then shook in 100% ethanol for about 5 h to remove chlorophyll. Subsequently, the samples were examined under an optical microscope.

### Drought Stress and Water Loss Experiments

To apply drought stress, the protocol described by Sakamoto et al. (2004) was followed with minor modifications. Seedlings were grown under well-watered conditions for four weeks, and then deprived of water for 2–3 weeks. Then, the plants were re-watered for over three days and photographed. For water-loss assays, rosette leaves were collected from 4-week-old plants as test samples. The samples were weighed immediately on a piece of paper, and then placed on the laboratory bench (relative humidity: 40%–50%; 22°C–23°C). The weight lost by each sample at a set of pre-assigned time points was recorded.

### Stomatal Movement

*Arabidopsis thaliana* plants were grown under a 10 h photoperiod (100 μmol m^-2^s^-1^ light), where the light and dark period temperatures were 22 and 20°C, respectively. The stomatal aperture assay was performed using fully expanded young leaves of 4-week-old plants. To characterize stomatal opening, detached rosette leaves were first floated adaxial side up for 2.5 h in the dark in 10 mM KCl, 7.5 mM iminodiacetic acid, 10 mM Mes-KOH (pH 6.15). Subsequently, either 50 μM ABA or solvent control (ethanol) was added, then the treated leaves were illuminated for 2.5 h. For the stomatal closure assay, initially leaves were floated in the solution containing 20 mM KCl, 1 mM CaCl_2_, 5 mM Mes-KOH (pH 6.15) for 2.5 h in the light, then either ABA (1, 10, or 50 μM) or solvent control (ethanol) was added. In both cases, abaxial epidermal strips were peeled off after 2.5 h incubation (with ABA/solvent control ethanol) and photographed immediately. Stomatal aperture widths were estimated from the captured digital images using ImageJ v1.47^5^ software.

### Electrophysiology

*Arabidopsis thaliana* guard cell protoplasts were isolated following the Zhang et al. (2008) method. K^+^ currents were recorded following Li et al. (2016) with slight modifications. The bath solution contained 4 mM MgCl_2_, 10 mM MES-Tris (pH 5.6), 1 mM CaCl_2_, 10 mM K-Glu, and the osmolarity was adjusted to 500 mOSM with sorbitol for measuring whole-cell channel K^+^ currents. The pipette solution contained 20 mM KCl, 10 mM HEPES-Tris (pH 7.8), and 80 mM K-Glu, and the osmolarity was adjusted to 550 mOSM, and also fresh ATP (5 mM Mg-ATP) was added before use. Axopatch-200B amplifier (Axon Instruments, United States) connected to a computer via an interface (TL-1 DMA Interface; Axon Instruments) was used to achieve whole-cell configuration, and the holding potential was set as –60 mV. The whole-cell currents were recorded 5 min later. The test voltage steps were from –200 mV to –40 mV with +20 mV increments, and each test voltage has 4.9-s duration. For the ABA treatment, 50 μM ABA was added to the bath solution after the whole-cell configuration was achieved. The whole-cell currents were recorded 10 min later. Guard cell anion currents were recorded as described by (Acharya et al., 2013). Briefly, the bath solutions contained 2 mM MgCl_2_, 30 mM CsCl, 10 mM MES-Tris (pH 5.6), and 1 mM CaCl_2_. The pipette solutions contained 150 mM CsCl, 2 mM MgCl_2_, 6.7 mM EDTA, 3.35 mM CaCl_2_, and 10 mM HEPES-Tris (pH 7.5). Osmolarity of the solutions was adjusted to 480 and 500 mOSM for bath and pipette solutions, respectively, with sorbitol. ATP (10 mM Mg-ATP) and GTP (10 mM) were added to the pipette solution before use. The holding potential was +30 mV. The voltage steps were applied from -145 to +35 mV with +30 mV increments, and each test voltage has a 60-s duration. For the ABA treatment, guard cell protoplasts were treated with 50 μM ABA for at least 1 h before recording.

### RT-PCR and qRT-PCR

Total RNA was isolated using the TRIzol reagent (Roche, Switzerland), treated with RNase-free DNase I (Invitrogen, United States) and reverse transcribed using oligo-dT primers and SuperScript^TM^ III Reverse Transcriptase (Invitrogen, United States). The resulting cDNA was used as template in both RT- and qRT-PCRs. The former employed the *CML20*-specific primer pair CML20-RTF/-RTR (**Table 1**) and reference reactions were primed by ACTIN2-RTF/-RTR (**Table 1**), to amplify the gene *ACTIN2* (At3g18780). For the qRT-PCRs, leaves and epidermal strips derived from four-week old plants were exposed to 50 μM ABA for 6 h. All reactions were performed in triplicates using FastStart Universal SYBR Green master mix (Roche, Switzerland), and *ACTIN2* was used as the reference. The *CML20*-specific primer pair CML20-QF/-QR and the *ACTIN2* pair ACTIN2-QF/-QR (**Table 1**) were used for the qRT-PCR assays. The abundance of transcript of a set of known stress-responsive genes was also assayed by qRT-PCR using cDNA synthesized from seedlings (two-week old) that were harvested 6 h after exposure to 50 μM ABA. The gene/primer pair combinations were *RAB18* (*At5g66400*)/RAB18F/R, *COR47* (*At1g20440*)/ COR47F/R, *MYB2* (*At2g47190*)/MYB2F/R, *ERD10* (*At1g20450*)/ERD10F/R, *KIN1* (*At5g15960*)/KIN1F/R, *RD29A* (*At5g52310*)/RD29AF/R, *KIN2* (*At5g15970*)/ KIN2F/R, and *APX2* (*At3g09640*)/APX2F/R. All primer sequences are given in **Table 1**.

### Fluorescent Imaging of ROS Production

Reactive oxygen species production in guard cells was detected by loading abaxial epidermal strips with H_2_DCF-DA (Zhang et al., 2011). Epidermal strips were peeled from detached leaves from plants, and floated in 10 mM KCl, 7.5 mM iminodiacetic acid, 10 mM Mes-KOH (pH 6.15) for 2 h to induce stomatal opening. Then, the epidermal strips were transferred to 10 mM Tris HCl, 50 mM KCl (pH 7.2) containing 50 mM H_2_DCF-DA, and held in the dark for 10 min. Unincorporated H_2_DCF-DA was removed by rinsing three times in double distilled water. The guard cells were then subjected to laser scanning confocal microscopy, and treated with either 50 μM ABA or solvent control before data collection. The fluorescence of H_2_DCF was captured by imaging at 2.5 min intervals over 25 min. ZEN software^6^ (2012, blue edition) was used to quantify the data. The change in fluorescence intensity at each time point was calculated in the form of a percentage of its initial intensity.

## Results

### *CML20* Encodes a CAM-Like Protein with the Ability to Bind Ca^2+^

**FIGURE 1:**
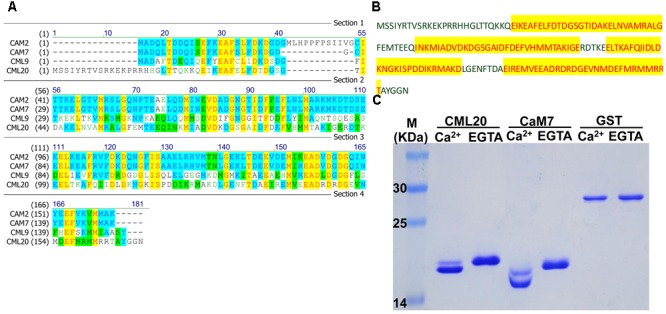
**CML20, a calmodulin-like protein, is able to bind Ca^2+^. (A)** Amino acid sequence alignment between CML20 and its homologs CAM2, CAM7, and CML9. **(B)** The amino acid sequence of CML20. The Ca^2+^-binding EF-hand motifs are highlighted in yellow. **(C)** The SDS-PAGE mobility shift assay showing that CML20 and CaM7 bind to Ca^2+^. GST was used as a negative control. The left-hand lane contains a protein size marker.

The predicted *CML20* product was a CAM-like protein consisting of 169 aa residues. The structure of CML20, according to InterPro^7^, contains four EF-hand domains (**Figure 1B**), as seen in numerous Ca^2+^ binding proteins (**Figure 1A**) such as CAM2, CAM7 and CML9 (Fabienne et al., 2008; Garrigos et al., 1991; McCormack et al., 2005). The SDS-PAGE mobility shift assay revealed that CML20 migrated faster in the presence of free Ca^2+^ than in the presence of Ca^2+^ chelator EGTA (**Figure 1C**). Like CML20, we also observed similar result for CAM7 (Garrigos et al., 1991). Our findings suggested that CML20 could bind Ca^2+^ *in vitro* and thus potentially acts as a Ca^2+^ sensor in plant.

### Expression Profiling of *CML20*

**FIGURE 2:**
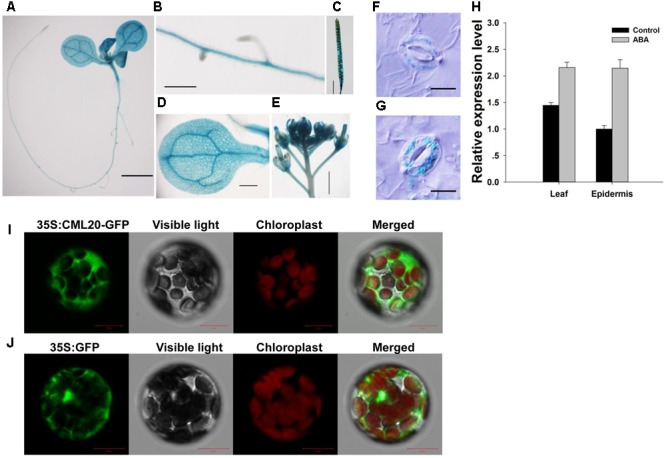
**The site of expression of *CML20*. (A–G)** GUS staining of various tissues in *A. thaliana* plants harboring *pCML20::GUS*. **(A)** The whole seedling. Bar: 2 mm, **(B)** the root. Bar: 0.5 mm, **(C)** the silique. Bar: 3 mm, **(D)** the leaf. Bar: 1.5 mm, **(E)** the Inflorescence. Bar: 1.5 mm, all without abscisic acid (ABA) treatment, **(F)** a guard cell in plants not exposed to ABA treatment. Bar: 10 μm, **(G)** a guard cell in plants 6 h after exposure to 50 μM ABA treatment. Bar: 10 μm. **(H)** Transcript levels of CML20 in leaves and epidermis exposed to 50 μM ABA treatment for 6 h or solvent control. Error bars show the SE (*n =* 3). **(I,J)** Transient expression of GFP and *CML20-GFP* in mesophyll protoplasts showing their sub-cellular localization **(I)** *p35S::CML20-GFP* and **(J)** *p35S::GFP*. Bar: 10 μm.

As deduced from the sites of GUS production in transgenic plants harboring *pCML20::GUS*, expression of *CML20* was observed in the root, leaf, inflorescence and silique (**Figures 2A–G**). *CML20* expression was also induced in the guard cells of plants that were exposed to ABA (**Figures 2F,G**). qRT-PCR result also supported that *CML20* was induced in response to ABA treatment in the leaf and epidermis, which contains a large number of guard cells (**Figure 2H**). All of these findings suggested the possible involvement of CML20 in the ABA-mediated regulation of stomatal movement. When mesophyll protoplasts were transformed with *p35S::CML20-GFP*, GFP signal was detected exclusively in the cytoplasm (**Figure 2I**), but mesophyll protoplasts transformed with *p35S::GFP* empty vector control showed GFP signal dispersed throughout the protoplast (**Figure 2J**). These findings indicate that CML20 is a cytosol-localized protein.

### *cml20* Mutant Plants Were Hypersensitive to ABA-Regulated Stomatal Movement and Show a Greater Tolerance to Drought Stress

**FIGURE 3:**
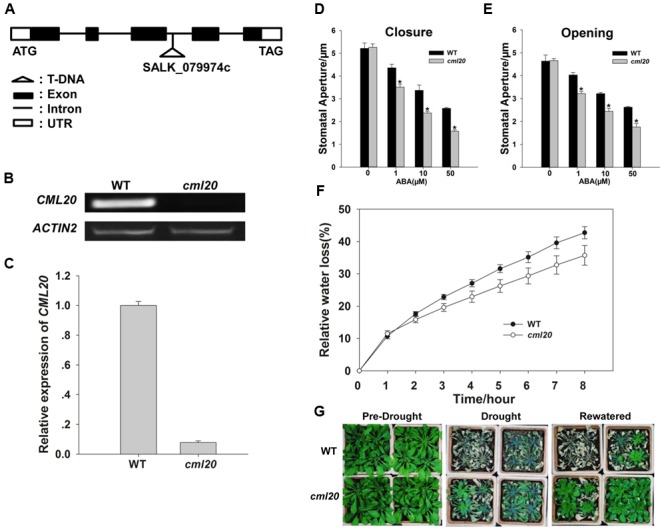
**Stomatal movement of the *cml20* mutant is hypersensitive to ABA treatment, making the plant more tolerant to drought stress. (A)** The T-DNA insertion site in the third intron of *CML20*. **(B,C)** *CML20* is not transcribed in the *cml20* mutant, as shown by **(B)** RT-PCR analysis and **(C)** qRT-PCR analysis. Error bars represent the SE (*n* = 3). The *ACTIN2* was used as an internal control. **(D,E)** The stomatal movement assay: **(D)** ABA induction of stomatal closure, **(E)** ABA inhibition of stomatal opening. At least 60 stomata were measured for each genotype per replication. ^∗^means differ significantly (*P* < 0.05). Error bars represent the SE (*n* = 3) from three independent experiments. **(F)** The rate of water loss from detached leaves of WT and the *cml20* mutant. Error bars represent the SE (*n* = 3). **(G)** The *cml20* mutant was more tolerant to drought stress than WT. The experiment was repeated three times with similar results.

Transcriptional analysis of the *cml20* mutant (**Figure 3A**) was carried out using both RT- PCR and qRT-PCR which confirmed the absence of *CML20* transcript (**Figures 3B,C**). Given that *CML20* was clearly up-regulated by ABA in the guard cells, the response of *cml20* guard cells was of interest to study its role in ABA signaling. Stomatal aperture was indistinguishable between the mutant and WT when plants were not treated with ABA, but in the presence of ABA (1, 10, or 50 μM), the mutant stomata showed smaller aperture than the WT (**Figures 3D,E**). The results indicated that *cml20* stomata were hypersensitive to both ABA-promoted stomatal closure as well as the ABA-inhibited stomatal opening, and thus to minimize the water loss to increase the plant drought tolerance. This hypothesis was supported by the observed results of both the detached leaf assay and the whole plant response to drought stress as well (**Figures 3F,G**).

### *CML20* Over-Expression Reduces the Sensitivity of Stomatal Movement to ABA and Has a Negative Effect on Drought Stress Tolerance

**FIGURE 4:**
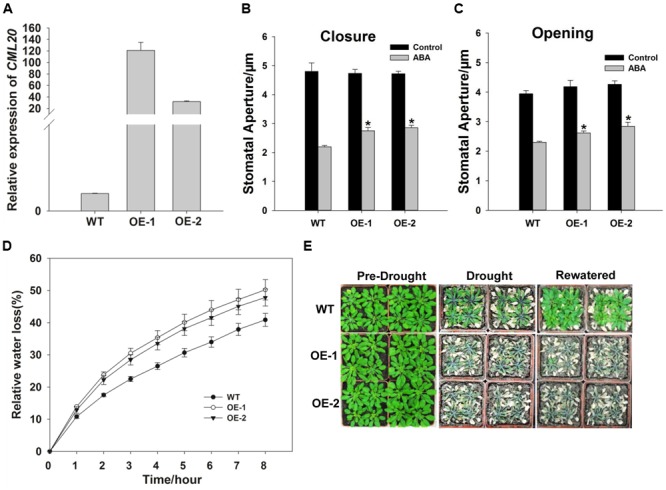
**The *CML20* over-expression lines OE-1 and -2 show hypersensitivity to drought stress. (A)** *CML20* transcript abundance in WT and the two over-expression lines (qRT-PCR assay). *ACTIN2* was used as the internal control. Error bars represent the SE (*n* = 3). **(B,C)** Stomatal movement in WT, OE-1 and OE-2 under control (without ABA) and 50 μM ABA conditions. At least 60 stomata were measured for each genotype per replication ^∗^: means differ significantly (*P* < 0.05), Error bars represent the SE (*n* = 3) from three independent experiments. **(D)** The rate of water loss from detached leaves of WT and the *CML20* over-expression lines. Each data point represents the mean Error bars represent the SE (*n* = 3). **(E)** The appearance of WT and the *CML20* over-expression lines grown under drought stress. The experiment was repeated three times with similar results.

qRT-PCR result revealed that *CML20* over-expressing lines, OE-1 and -2 both produced more *CML20* transcript than WT (**Figure 4A**). Stomatal movement in these lines was less affected by ABA than in the WT (**Figures 4B,C**). As predicted, the rate of water loss from detached leaves of the two OE lines was higher than WT leaves (**Figure 4D**), and the OE-1 and OE-2 plants were more sensitive to drought stress (**Figure 4E**). In addition, *cml20*/*CML20* complementation lines (C-1 and C-2) showed similar phenotype for both ABA regulation of stomatal movement and drought stress resistance compared to WT (Supplementary Figure S1). These findings provided additional support that CML20 functions as a negative regulatory signaling component in response to both ABA and drought stress.

### CML20 Is Involved in the ABA Regulation of Guard Cell Ion Channels

**FIGURE 5:**
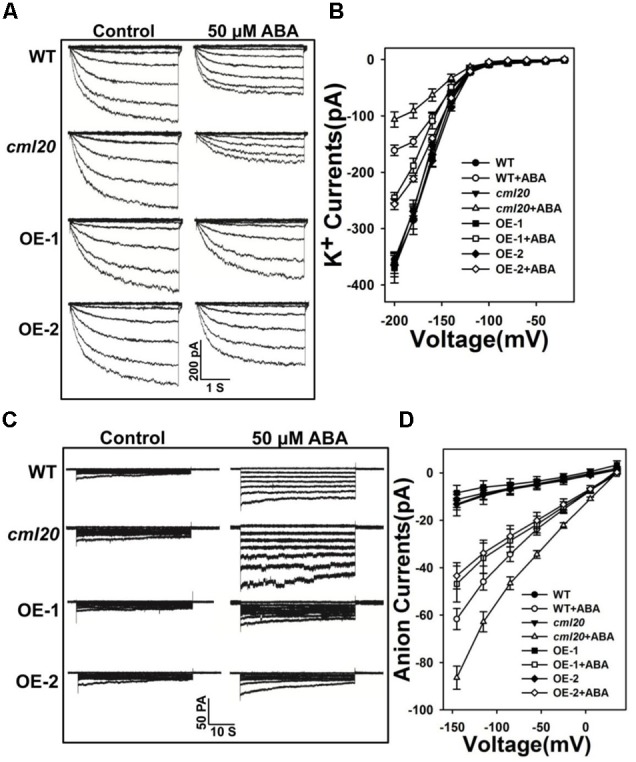
**CML20 is involved in the ABA regulation of inward K^+^ and slow-type anion channels. (A)** Patch-clamp whole cell recordings of the inward K^+^ current in guard cell protoplasts isolated from WT, *cml20* and the *CML20* over-expression lines OE-1 and -2, either in the presence or absence of 50 μM ABA. **(B)** Current/voltage relationships of whole cell K^+^ currents. The number of guard cells measured were: WT (8), WT+ABA (9), *cml20* (7), *cml20*+ABA (8), OE-1 (7), OE-1+ABA (11), OE-2 (11), OE-2+ABA (12). Error bars represent the SE. **(C)** ABA (50 μM) activation of slow anion currents in guard cell protoplasts of WT, *cml20*, OE-1, and OE-2. **(D)** Current/voltage relationships of whole cell slow anion currents. The number of guard cells measured were: WT (9), WT+ABA (7), *cml20* (9), *cml20*+ABA (11), OE-1 (6), OE-1+ABA (7), OE-2 (11), OE-2+ABA (10). Error bars represent the SE.

Stomatal closure relies on anion efflux via channels that are activated by ABA, which also simultaneously inhibits the influx of K^+^. The patch clamp assay was conducted to test whether ABA-modulated currents depended on *CML20.* Without ABA treatment, we did not observe any difference in either the K^+^ or the anion currents between WT, *cml20* and the two OE lines. In contrast, in response to ABA treatment, the mutant’s guard cells showed hypersensitivity to ABA inhibition of K^+^ currents and ABA activation of anion currents, while the over-expression of *CML20* impaired them both (**Figures 5A–D**). These results coincided with the performance of stomatal movement in response to ABA treatment. The conclusion was that CML20 functioned as a negative regulatory signaling component for ABA regulation of stomatal movement, partly via its regulatory effect on ion channels activity in guard cells.

### Loss of Function of CML20 Affects the Transcription of Stress-Responsive Genes

**FIGURE 6:**
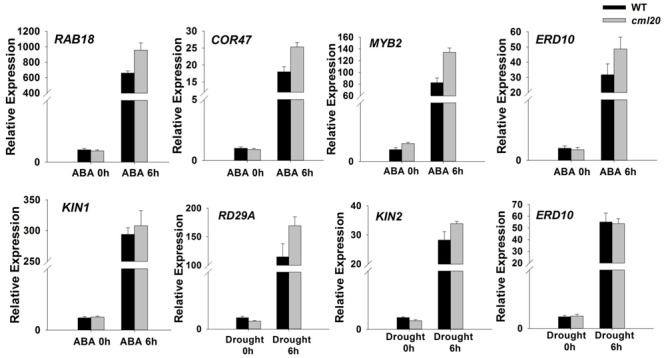
**The transcription of the indicated stress-responsive genes was altered in the *cml20* mutant.** *RAB18, COR47, MYB2* and *ERD10* were up-regulated by exposure to 50 μM ABA for 6 h. *RD29A* and *KIN2* were up-regulated by exposure to drought stress for 6 h. *ACTIN2* was used as the internal control. Error bars represent the SE (*n* = 3).

Transcriptional (qRT-PCR) profiling showed that in *cml20*, certain ABA-inducible genes (*ERD10, RAB18, COR47*, and *MYB2*) were up-regulated by exposure to ABA for 6 h (**Figure 6**). The imposition of drought stress also induced the transcription of an ABA-independent gene *RD29A* in the *cml20* mutant (**Figure 6**). The aforementioned genes are up-regulated in response to abiotic stresses also their transcript level could be correlated with sensitivity to a specific stress. These findings not only implicate that CML20 negatively regulates transcription of these above indicated genes but also suggest that CML20 is also a regulator of the ABA and drought stress signaling pathways.

### ROS Production in *cml20* Guard Cells Is Enhanced by Exposure to ABA

**FIGURE 7:**
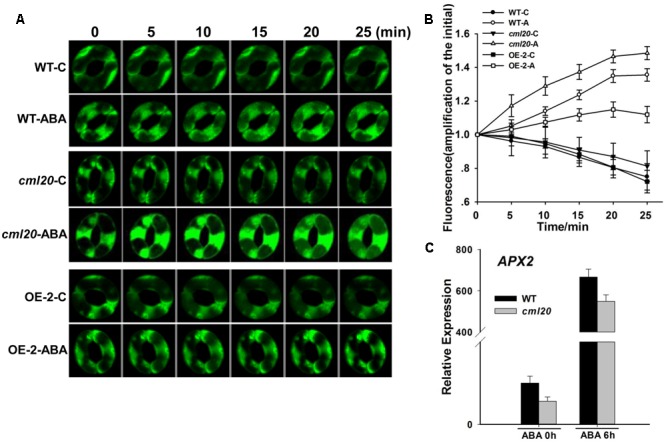
**Reactive oxygen species (ROS) production is elevated in the guard cells of *cml20* plants exposed to ABA. (A)** H_2_DCF fluorescence in WT, *cml20* and OE-2 imaged at 5 min intervals over 25 min. At least ten guard cells were measured. **(B)** H_2_DCF-DA staining revealed a higher level of ROS production in *cml20* than in WT, and a lower level in OE-2. Error bars represent the SE. **(C)** The transcription of *APX2* was down-regulated in *cml20*. Error bars represent the SE (*n* = 3).

The ABA treatment resulted in a greater increase of ROS production in the guard cells of *cml20* than in those of WT (**Figures 7A,B**). A qRT-PCR based assay of the transcription of a gene known to be involved in ROS elimination showed that *APX2* (encoding an H_2_O_2_ scavenger) was down-regulated in *cml20* whether or not the plants were exposed to ABA (**Figure 7C**). These above findings suggest that CML20 may play a positive role in guard cell ROS removal; in its absence, the down-regulation of *APX2* could have compromised the plant’s ability to control the accumulation of ROS.

## Discussion

As shown by the SDS-PAGE mobility shift assay (**Figure 1**), CML20 demonstrated an ability to bind Ca^2+^ *in vitro*. Many Ca^2+^ sensors have been implicated as signaling components in the abiotic stress response. An example is the rice gene *OsCaM1-1*, which is induced by exposure to high temperature; its product enhances thermo tolerance when the gene is constitutively expressed in *A. thaliana* (Wu et al., 2012). Transcription factors such as *AtCAMTA1, -A2* and -*A3* are known to participate in response to low temperature stress (Kim et al., 2013). The product of *AtCAMTA1* is also involved in the drought stress response (Pandey et al., 2013). Despite being one of the largest Ca^2+^ sensor families, few CMLs have been identified yet that participate in the abiotic stress response: these comprise the *A. thaliana* proteins CML9, 10, 18, 24, and 42, as well as the rice protein OsMSR and ShCML44 from *Solanum habrochaites* (Delk et al., 2005; Yamaguchi et al., 2005; Fabienne et al., 2008; Xu et al., 2011; Vadassery et al., 2012; Cho et al., 2015; Munir et al., 2016). Here, the current work demonstrated that AtCML20 is negatively involved in guard cell ABA signaling during plant drought response.

A consistent implication emerged from the present set of experiments was that CML20 acted as a negative regulator of ABA and drought stress responses in plants. Stomatal movement was more sensitive to the ABA treatment in the *cml20* mutant than in the WT (**Figure 3**). In addition, OE lines with higher expression of *CML20* showed less sensitivity to ABA-mediated stomatal movement, which might have caused OE plants to be lower ability of drought tolerant. In plants, stomatal closure represents an early response to drought stress, and aids for the maintenance of the plant’s internal osmotic environment (Verslues et al., 2006). Thus, the action of CML20 to inhibit stomatal movement has a negative effect on the plant’s tolerance to drought stress. Nevertheless, WT plants treated with ABA showed 100% increase of *CML20* transcript (**Figure 2H**). A possibility is that the function of CML20 during an episode of drought stress is to enforce stomata to remain in a partial open state, a physiological condition that would allow the plant to exchange gas.

Interestingly, we further found that *CML20* had roles in ABA regulating ion channels. The results from patch clamp assay indicated that both K^+^_in_ channels and anion channels were influenced in loss-of-function mutant and OE lines in response to ABA (**Figure 5**). Some CDPKs, as Ca^2+^ sensors like CMLs, have been reported to regulate guard cell SLAC1 channel’s activity (Geiger et al., 2010). We also explored the molecular mechanism to define the function of CML20 in ABA signaling.

ABA is a critical regulator of the abiotic stress response, which acts via complex signaling networks. From the qRT-PCR analysis, it was obvious that the loss-of-function of *CML20* affected the transcription of a number of ABA-inducible genes in mutant plants treated with ABA (**Figure 6**), which in turn may have been responsible for the mutant’s enhanced level of drought stress tolerance. By acting as a negative regulator, CML20 likely limits the extent of the responsiveness of these genes to ABA; it also negatively regulates the transcription of *RD29A*, a gene which responds to drought stress, in an ABA- independent manner (Yamaguchi-shinozaki and Shinozaki, 2006).

Fluorescent probes demonstrated that the *cml20* mutant generated more ROS than WT under ABA treatment (**Figures 7A,B**), consistent with the negative role of CML20 in stomatal movements and drought response. ROS is generated by NADPH oxidases (Respiratory Burst Oxidase Homologs, RBOHs), and the enzyme AtRBOHD and AtRBOHF are mainly expressed in guard cells and could be up-regulated by ABA. In the ABA signal pathway, OST1 is released by ABA from PP2C to activate AtRBOHF through directly phosphorylation (Kwak et al., 2003; Sirichandra et al., 2009; Shang et al., 2016). In another way, the elimination of ROS is achieved by a range of antioxidants and enzymes. The gene *APX2*, which encodes a cytosolic ascorbate peroxidase (a ROS scavenger), had a lower expression level in *cml20* than WT whether or not the plants were exposed to ABA (**Figure 7C**). A reduced presence of the ROS scavenging enzyme APX2 in ABA-treated *cml20* mutant plants was predicted from the lowered abundance of *APX* transcript in *cml20* compared to that in WT, may have allowed for a greater accumulation of ROS, which in turn could have enhanced the transduction of the ABA signal.

Meanwhile the prominent ROS species H_2_O_2_, which is induced by ABA in the guard cells, can activate Ca^2+^ channels and regulate stomatal movement (Pei et al., 2000; Mustilli et al., 2002), inversely, Ca^2+^ could also bind and activate RBOH for ROS generation (Ogasawara et al., 2008; Kimura et al., 2012). Both Ca^2+^ and ROS are important signal integrators in plants (Murata et al., 2015). Given its proven Ca^2+^ binding ability, CML20 may play roles both in Ca^2+^ and ROS signal pathways to regulate stomatal aperture in plants either exposed to exogenous ABA or subjected to drought stress.

**FIGURE 8:**
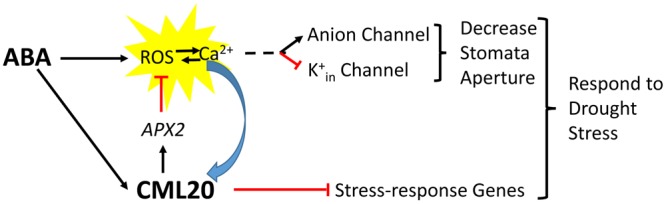
**A proposed model for the signaling role of CML20 in the guard cell in response to ABA**.

The CML20 may serve as a fine regulator for cytosol ROS homeostasis in guard cell ABA signaling. Previous studies have demonstrated that the guard cell anion channels (*SLAC1*) and K^+^_in_ channels are regulated by ROS (Köhler et al., 2003; Joo et al., 2005; Sierla et al., 2016), and the APX2 serves as a ROS scavenger to reduce ROS concentration in cytosol (Fryer et al., 2003). Here we show that, the *cml20* mutants were with lower APX2 transcripts and higher ROS (**Figure 7**), so the CML20 may serve as opposite factor with ABA to avoid too much ROS accumulation, and to keep appropriate stomatal aperture under finely balanced control. In addition, CML20 could also be the negative regulator of ABA and drought via other plant abiotic stress responsive gene mediated pathways (**Figure 8**). Besides ROS, Ca^2+^ and ion channels, there may be other factors along with CML20 are involved in the signaling cascades that play critical roles in response to drought stress and ABA treatment. Follow-up research will attempt to identify the targets of CML20, so as to further elucidate the signaling pathways used by plants that are exposed to abiotic stress.

## Author Contributions

WZ designed the experiments; XW, CL, and ZQ performed the experiments with assistance from HL; WZ, XW, CL, ZQ, and BA analyzed and discussed the results; WZ, XW, CL, and ZQ wrote the manuscript.

## Conflict of Interest Statement

The authors declare that the research was conducted in the absence of any commercial or financial relationships that could be construed as a potential conflict of interest. The reviewer GM and handling Editor declared their shared affiliation, and the handling Editor states that the process nevertheless met the standards of a fair and objective review.

## Footnotes

1 http://abrc.osu.edu/

2 http://signal.salk.edu/tdnaprimers.2.html

3 http://www.ebi.ac.uk/interpro/

4 http://www.mn-net.com

5 https://imagej.nih.gov/ij/

6 http://www.zeiss.com.cn/microscopy/zh_cn/downloads/zen.html

7 http://www.ebi.ac.uk/interpro/
