# Supplementary material for: CML20, an Arabidopsis Calmodulin-like Protein, Negatively Regulates Guard Cell ABA Signaling and Drought Stress Tolerance
Source: Front Plant Sci. 2017 May 23;8:824. doi: 10.3389/fpls.2017.00824 (PMC5445667; doi:10.3389/fpls.2017.00824)
Supplement: Supplementary file 1 [file Image_1.PDF]

Supplementary Material

CML20, an *Arabidopsis* Calmodulin-like Protein, Negatively Regulates Guard Cell ABA Signaling and Drought Stress Tolerance

Xiaomeng Wu, Zhu Qiao, Huiping Liu, Biswa R. Acharya, Chunlong Li and Wei Zhang

***Correspondence**： Chunlong Li, lichunlong216@126.com;
Wei Zhang, weizhang@sdu.edu.cn

Supplementary Figure

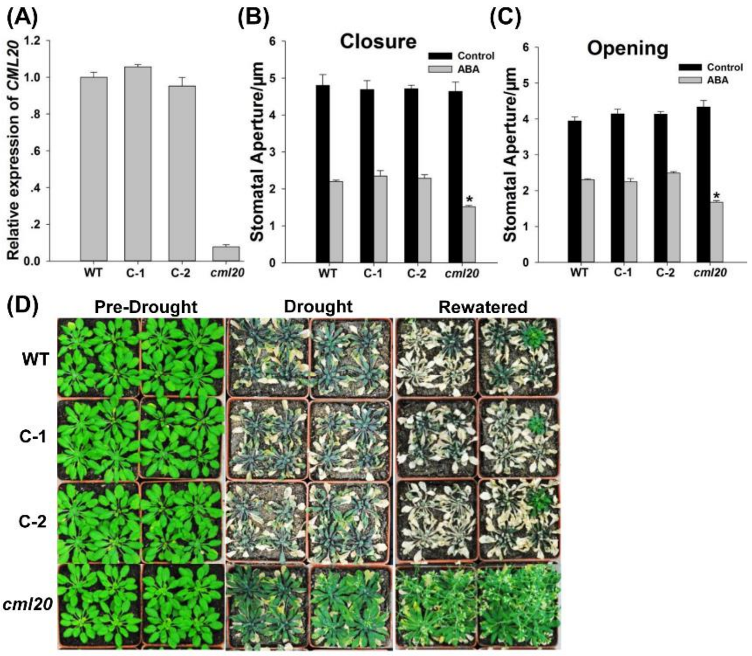

Supplementary Fig. 1. The phenotype of *cml20*/*CML20* complementary lines C-1 and C-2. **(A)** *CML20* transcript in WT and the two complementary lines (qRT-PCR assay). *ACTIN2* was used as the internal control. Error bars represent the SE (*n*=3). **(B, C)** Stomatal movement in WT, C-1 and C-2 either in the presence or absence of 50 μM ABA. At least 60 stomata were measured for each genotype per replication *: means differ significantly (P<0.05), Error bars represent the SE (*n*=3) from three independent experiments. **(D)** The phenotypic appearance of WT, complementary lines and the *cml20* mutant grown under drought stress. The experiment was repeated three times with similar results.
